# Detection and Typing of Human Papilloma Virus by Multiplex PCR with Type-Specific Primers

**DOI:** 10.5402/2012/186915

**Published:** 2012-03-01

**Authors:** Francisco Romero-Pastrana

**Affiliations:** FRP Genética, 115A Ote 1417-1, 72520 Puebla, PUE, Mexico

## Abstract

The primary underlying cause of cervical cancer is infection with one or more high-risk (HR) types of the human papilloma virus (HPV). Detection and typing of HPV have been commonly carried out by PCR-based assays, where HPV detection and typing are two separate procedures. Here, we present a multiplex PCR-based HPV typing assay that detects 20 HPV types (15 HR, 3 probably HR and 2 low risk) using type-specific primers and agarose gel electrophoresis. 46 cervical, urethral, and biopsy samples were analyzed by both Multiplex PCR and PGMY09/11 consensus PCR, and results were compared. 611 samples were further analyzed by Multiplex PCR, 282 were positive for HR HPV, and 101 showed multiple HR HPV infections. The relatively ease and economic accessibility of the method and its improved ability to detect high-risk HPV types in multiple HPV-infected samples make it an attractive option for HPV testing.

## 1. Introduction

 Cervical cancer is the second most common cancer in women worldwide [1] and is the most common cancer in women from low-income countries, where an estimated 80% of cases occur [2]. 16,000 cases of cervical cancer are newly detected every year in Mexico, resulting in a high incidence rate (50 cases per 100,000 women) [3, 4]. The primary underlying cause of cervical cancer is infection with one or more high-risk (HR) types of the human papilloma virus (HPV) [5–10]. 15 HR types (16, 18, 31, 33, 35, 39, 45, 51, 52, 56, 58, 59, 68, 73, and 82) have been proposed, including 3 types (26, 53, and 66) that should be considered probably carcinogenic [11, 12].

Detection and typing of HPV have been commonly carried out by PCR-based assays, where HPV DNA is amplified by consensus primers and then typed by restriction enzyme analysis (RFLP), hybridization with type-specific probes, or direct sequencing of the amplicons, among the most common methods [13]. Recently, methods that use multiplex PCR amplification with type-specific primers have been reported, where detection and typing are deducted from the amplification pattern of capillary electrophoresis [14].

Here, we present a multiplex PCR-based HPV typing assay that detect 20 HPV types (15 HR), 3 probably HR and 2 low risk (LR) using type-specific primers and agarose gel electrophoresis.

## 2. Materials and Methods

### 2.1. Sample Preparation

611 samples of cervical (232) and urethral (164) scrapes and paraffin-embedded tissue biopsies (215) submitted for HPV assessment were collected for Multiplex PCR HPV analysis. A subset of 46 cervical, 16 urethral, and 21 tissue biopsies were randomly selected for additional analysis with PGMY09/11 consensus primer PCR. DNA extraction of samples was performed using the Dneasy Tissue Kit (Qiagen, Germany), following manufacturer's instructions.

### 2.2. Primers Design

DNA sequence files for HPV types 6, 11, 16, 18, 26, 31, 33, 35, 39, 45, 51, 52, 53, 56, 58, 59, 66, 68, 73, and 82 were obtained from Genbank (http://www.ncbi.nih.gov/genbank/). Primers were designed for each HPV type, and unique specificity was confirmed by BLAST analysis (http://www.ncbi.nlm.nih.gov/BLAST/). Primer selection for each reaction tube mix was carried out *in silico* [15] and experimentally to ensure primer compatibility, and a primer pair specific for *β*-globin was included as positive control [16]. Sequences of primers included in each reaction mix, with predicted amplification product size and digestion product sizes with indicated restriction enzymes are shown in Table 1.

### 2.3. Multiplex PCR

The QIAGEN Multiplex PCR kit (Qiagen, Germany) was used in all Multiplex PCR reactions, following manufacturer instructions. Each PCR was carried out in a DNA thermal cycler (MaxyGene Gradient Thermal Cycler, Axygen Scientific, USA) with the following conditions: initial denaturing step at 95°C for 15 min, 10 cycles of 30 s at 94°C, 90 s at 65°C, and 90 s at 72°C, followed by 30 cycles of 30 s at 94°C, 90 s at 63°C, and 90 s at 72°C, with a final extension at 72°C for 10 min. PCR products were analyzed by electrophoresis on a 2% agarose gel stained with ethidium bromide, band sizes were estimated by comparison with a 100 bp molecular weight marker (GeneRuler 100 bp DNA Ladder, Fermentas International, Canada), and gels were photographed in a UV transilluminator (UVP, USA) with a Canon PowerShot A60 digital camera (Canon, USA). HPV type was assigned based on the amplification pattern. In cases where band interpretation was not clear, an additional PCR amplification with specific primers was performed to confirm. Selected PCR amplified fragments were cloned into pGem-T vector (Promega, USA), each cloned product was sequenced with universal forward and reverse primers to confirm fragment identity. Additionally, selected amplified fragments were digested with restriction enzymes AluI, HaeIII, RsaI, or MspI (New England Biolabs, USA), and digestion patterns were observed in a 2% agarose gel to also confirm fragment identity.

### 2.4. PGMY09/11 Consensus PCR

HPV consensus PCR was performed using primers PGMY09/PGMY11 designed to amplify a fragment of the HPV L1 gene of approximately 450 bp as previously described [17]. HPV genotype was assigned by sequencing of amplified fragments using primers PGMY11.

## 3. Results and Discussion

Polymerase Chain reaction (PCR) with consensus primers can potentially detect most mucosal HPV types [18]. There are several consensus primers described, GP5/6 and their improved GP5+/6+ [19, 20], SPF [21], My09/11 and their improved PGMY09/11 [17, 18, 22], L1C1 with L1C2 and L1C2M [23], pU-1M/pU-2R and their enhanced pU-1M-L and pU-2R-N [24]. Typing of amplified fragments is usually performed by different techniques such as, hybridization to specific probes [25], restriction fragment length polymorphisms [26, 27], or direct DNA sequencing [28–30].

**Figure 1:**
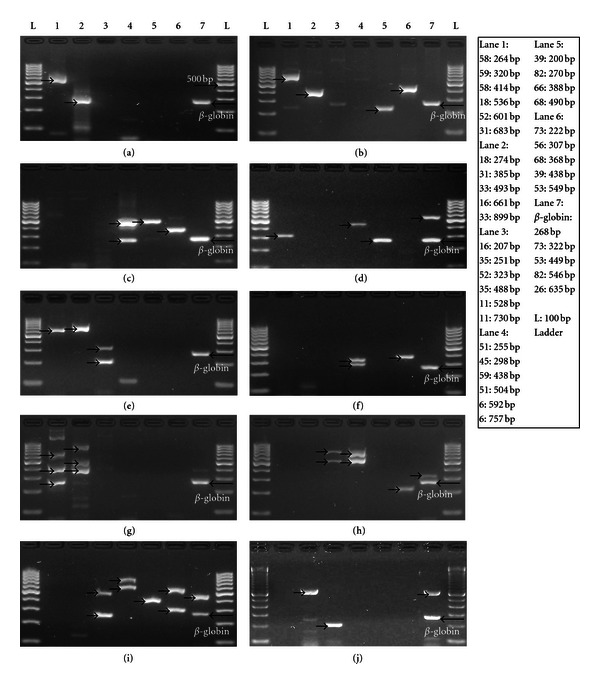
HPV detection and typing by Multiplex PCR. (a) Amplification pattern shows two bands in lanes 1 and 2 (arrows), consistent with the expected amplification pattern of HPV 18. (b) Four bands are observed in lanes 1, 2, 5, and 6 (arrows), consistent with HPV 31 and 39. (c) Bands in lanes 4, 5, and 6 (arrows), consistent with HPV 51 and 68. (d) Detection of HPV 59 and 82, (e) HPV 16 and 52, and (f) HPV 45 and 56. (g) Detection of HPV 31, 33, and 58, and (h) HPV 6, 11, and 73. (i) Five HR HPV types are observed: HPV 6, 35, 53, 56, and 66. (j) Detection of HPV 16 and 26, only one case of HPV 26 was detected in 611 samples.

In this report, we present an assay based in HPV DNA amplification with type-specific primers in a Multiplex PCR format to detect and type single or multiple HR HPV infections in samples of different sources. Primers specific to each of 15 high-risk, 3 probably high-risk, and 2 low-risk HPV types were included in seven independent Multiplex PCR reactions. Typing was assigned based on the amplification pattern. As a result of having specific primers, stringent PCR conditions can be set to increase the clarity of results by reducing the presence of amplification artifacts, and all HPV types in a sample are amplified by their specific primer pair. Also, detection and HPV typing are accomplished at the same time, without the need of an additional protocol for typing after HPV detection (see examples of HPV detection in Figure 1).

In order to evaluate the newly developed HPV detection assay, 83 samples (46 cervical and 16 urethral scrapes and 21 tissue biopsies) were analyzed with Multiplex PCR and PGMY09/11 consensus PCR. Positive high risk is determined when a HR or probable HR HPV type is detected, and negative high risk is determined when a LR HPV type or no HPV infection is detected (Table 2). 47 samples reported negative results by both Multiplex PCR (Multiplex) and by PCR with PGMY09/11 primers (PGMY), while no samples were reported positive by PGMY and negative by Multiplex. 19 samples were reported positive by both Multiplex and PGMY, but 17 samples were reported positive by Multiplex and negative by PGMY. Of those, 12 samples did not produce any amplified fragment (no detection) and 5 samples reported LR HPV types (failed to detect the HR HPV type also present in the sample). These results suggest that Multiplex PCR can detect HR HPV as well as PGMY PCR, and that Multiplex PCR can potentially detect HR HPV infections not reported by PGMY PCR, due to the presence of LR types that are preferentially amplified over HR types in multiple infections, as observed in 5 samples.

Particularly important is the capacity of detection of HPV multiple infections. A total of 611 samples (232 cervical and 164 urethral scrapes and 215 tissue biopsies) were analyzed by Multiplex PCR, including 83 samples mentioned above. 324 (53.03%) samples were negative for HR HPV, and 282 (46.15%) samples were positive for HR HPV. Only 5 (0.82%) samples (1 cervical and 4 paraffin-embedded tissue biopsies) failed to amplify the *β*-globin control gene and were reported as being not informative. 35% (101) of HR HPV positive samples had infections with two or more HR HPV types, representing 16% of the total number of samples analyzed. Detecting all HR HPV present in a sample is important in patient treatment to asses prevalence of infection and response to treatment.

Multiplex PCR HPV detection and typing are simple and potentially affordable. After DNA extraction and Multiplex PCR amplification, detection and typing of HPV are deduced from the amplification pattern observed in an agarose gel electrophoresis. This is particularly important in low-income countries. According to a study in Peru [31], simple, effective, and cost-efficient HPV testing is the best option for primary cervical screening. The entire cost in Mexico of the equipment and reagents for DNA extraction, amplification, agarose gel electrophoresis, and documentation is approximately $22,000 USD, and many research laboratories in Mexico already have all the necessary equipments.

## 4. Conclusions

Multiplex PCR HPV can detect single or multiple HR HPV infections in cervical and urethral scrapes and paraffin-embedded tissue biopsies. The relatively ease and economic accessibility of the method can potentially have an impact in HPV screening in low-income countries like Mexico, and its improved ability to detect high-risk HPV types in multiple HPV-infected samples makes it an attractive option for HPV testing.

## Figures and Tables

**Table 1:** Multiplex PCR primer list.

Name	Forward primer sequence	Reverse primer sequence	lane	size bp	R.E.	Digested fragment sizes
6-1	acgtggccttgtgcggtacagtc	agagacgagtcaggcaatgc	4	757	HaeIII	259, 250, 101, 72, 68, 7
6-2	tgtcccatctgcgcaccgaagac	cgtacactgtttgtgggcgcttc	4	592	AluI	366, 129, 97
11-1	agttccgtagatgccaagggca	tgcctcaggtgaggcccaatgc	3	528	RsaI	194, 172, 94, 68
11-2	tggtaccccctacacagggtgg	acagaatgttggacagggtcagg	3	730	HaeIII	433, 160, 137
16-1	ttaggcagcacttggccaacca	taatccgtcctttgtgtgagct	3	207	MspI	110, 97
16-2	actgcaatgtttcaggacccac	cgaagcgtagagtcacacttgc	2	661	MspI	405, 199, 57
18-1	tcgcgtcctttatcacagggcga	tgcccaggtacaggagactgtg	1	536	AluI	235, 200, 101
18-2	tccgtggtgtgcatcccagcag	cacttgtgcatcattgtggacc	2	274	RsaI	185, 48, 41
26-1	tggtatacaacgagtgtcagctcc	ggggcaatgatggccatgtcg	7	635	MspI	425, 210
31-1	aggcacggttggtgaatcggtc	tagatgctgagggtgcactacg	1	683	HaeIII	481, 202
31-2	catgaactaagctcggcattgg	tccaacatgctatgcaacgtcc	2	385	RsaI	233, 152
33-1	agcttagaggtgtggctttgtg	tgcagttagttgcagtacgtgc	2	493	RsaI	211, 145, 122, 15
33-2	tgacccacctacagctgcaatc	gggtgtgtacattatccacatcg	2	899	RsaI	615, 277, 7
35-1	ccaccaagtggttccaacgcag	tgtaggcgtgtagctgtgtagc	3	488	RsaI	216, 193, 79
35-2	gtcctgttggaaaccaacacgt	acacacagacgtagtgtcgcct	3	251	AluI	135, 81, 35
39-1	acacaaacggtgtattccgtgcca	tgtgcagttggagatttgggatcc	5	200	RsaI	129, 71
39-2	tgtgcagtaccagtgacggatcg	atttttggcgttgtgactctgtg	6	438	HaeIII	221, 217
45-1	ggacatcacacctaccgtggac	ctgtgaggtggacacacggacc	4	298	RsaI	126, 69, 59, 26, 18
45-2	acctgcacaattgcaacctggt	caactgccaggggtttcacgca	4	345	RsaI	179, 105, 61
51-1	aattgctggcaacgtacacgac	acacttgaacacctgcaacacg	4	255	RsaI	190, 51, 14
51-2	cctactccaggggttagtcgca	taaggagggcaactgcctagac	4	504	HaeIII	270, 234
52-1	cccaagtgtaacgtcatgcgtg	agggttgtttatagccgtgcac	3	323	AluI	215, 108
52-2	acctccgcagtgtccgtgggtg	aagagcggcctaagcactgcac	1	601	RsaI	143, 126, 96, 95, 66, 41, 34
53-1	ttgttcagtgtacggggctagc	gtgacgccattgcagttatcgcct	6	549	MspI	389, 160
53-2	ttctgcagtaagctatgagggcat	aaccactgtcgatttcggtgtt	7	449	MspI	270, 179
56-1	ctgggcactaggtcaaagcctgct	caaccacgcgtaaaagcactcat	6	307	AluI	278, 29
58-1	ggtagtaccccaccgtctgagg	agacgtgacattgccactgtca	1	414	MspI	289, 125
58-2	accagactccagagacaacacc	tcacctttgtcatcactggtcc	1	264	RsaI	165, 62, 25, 12
59-1	agacaccgttacatgagctgct	tcattctcggagtcggagtcag	1	320	AluI	213, 91, 16
59-2	tctaacgccatctgcagcaagg	acagtagtccactgacacgctg	4	438	HaeIII	339, 99
66-1	tgcggtagtatccttgggcagtg	tacaataagggctacacgccaa	5	388	RsaI	135, 131, 122
68-1	ggtactgcttggaacacgcctg	ggcccccagacatagggacctt	6	368	RsaI	307, 57, 4
68-2	gtcaaaaagacgcccctgcaccta	cacaccttagggtagggctacaa	5	490	HaeIII	329, 161
73-1	acaggctattagttgccaacgtc	ttcttaggtgtggcacttgtg	6	222	AluI	115, 62, 45
73-2	ggggtgggcaaaggtaggtagc	acaatccaggggcctctggtccga	7	322	RsaI	189, 108, 25
82-1	tgtccgtggacacctgcgacca	gtagttaaaggtgatgtggcaacc	7	546	RsaI	275, 215, 56
82-2	cccaaaaccaatacacgtgctgaa	aacatcctgttggtcgttgcca	5	270	HaeIII	189, 81
*β*-globin	gaagagccaaggacaggtac	caacttcatccacgttcacc	7	268		

**Table 2:** 83 samples analyzed by Multiplex PCR and PGMY consensus PCR.

Detection comparison of HR HPV	Multiplex PCR	
PGMY PCR	Positive	Negative
Positive	13 cervical	0 cervical
	4 urethral	0 urethral
	2 biopsies	0 biopsies

Negative	10 cervical	23 cervical
	2 urethral	10 urethral
	5 biopsies	14 biopsies
